# Levofloxacin or Clarithromycin-based quadruple regimens: what is the best alternative as first-line treatment for *Helicobacter pylori* eradication in a country with high resistance rates for both antibiotics?

**DOI:** 10.1186/s12876-017-0589-6

**Published:** 2017-02-15

**Authors:** Diogo Branquinho, Nuno Almeida, Carlos Gregório, José Eduardo Pina Cabral, Adriano Casela, Maria Manuel Donato, Luís Tomé

**Affiliations:** 1 Gastroenterology Department, Coimbra University Hospital, Praceta Prof. Mota Pinto, 3000-075 Coimbra, Portugal; 2 Gastroenterology Centre, Faculty of Medicine, Coimbra University, R. Larga, 3004-504 Coimbra, Portugal

**Keywords:** Eradication, First-line, *Helicobacter pylori*, Levofloxacin, Sequential therapy

## Abstract

**Background:**

*Helicobacter pylori* eradication rates in Portugal are declining, due to increased resistance of this bacterium to antimicrobial agents, especially Clarithromycin. Quadruple Levofloxacin-containing regimens could be an option for first-line treatment, but its efficacy should be evaluated as fluoroquinolone resistance is rapidly increasing.

Our aim was to compare the efficacy of Clarithromycin and Levofloxacin-based sequential quadruple therapies as first-line treatment options and determine factors associated with treatment failure.

**Methods:**

A total of 200 *Helicobacter pylori* infected patients were retrospectively included (female 57.5%; average age: 53.2 ± 15.7) and received either 10-day sequential therapy (Proton-Pump Inhibitor + Amoxicillin 1 g bid for 5 days and Proton-Pump Inhibitor + Clarithromycin 500 mg + Metronidazole/Tinidazole 500 mg bid/tid in the following 5 days; group A) or a 10-day modified sequential therapy with Levofloxacin 500 mg id instead of Clarithromycin (group B). Eradication was confirmed with urea breath test. Variables that could influence success rate were analyzed.

**Results:**

There were no differences between groups in terms of gender, age, smoking habits and indications for treatment. The eradication rate obtained with Clarithromycin-based sequential treatment was significantly higher than with Levofloxacin-based therapy (90%, CI95%: 84–96% vs. 79%, CI95%: 71–87%, *p* = 0.001). Using full-dose proton-pump inhibitor and high-dose Metronidazole in group A, and full-dose proton-pump inhibitor and prescription from a Gastroenterologist in group B were associated with eradication success.

**Conclusions:**

Ten-day Levofloxacin-based sequential treatment achieved inadequate efficacy rate (<80%) and should not be adopted as first-line therapy. Standard sequential therapy showed significantly better results in this *naïve* population. Using full-dose proton-pump inhibitor and higher doses of Metronidazole is essential to achieve such results.

**Electronic supplementary material:**

The online version of this article (doi:10.1186/s12876-017-0589-6) contains supplementary material, which is available to authorized users.

## Background

*Helicobacter pylori (H. pylori)* is a Gram negative, microaerophilic bacterium that is presumed to infect from one-third to over half of the world’s population [1]. It is known to be a major responsible for a significant number of gastric pathologies, but its role in extra-gastric diseases is also becoming well documented. *H. pylori* leads to an inflammatory state, induces molecular mimicry and alters the absorbance of several nutrients [2]. These discoveries are increasing physicians’ awareness to the need to eradicate this bacterium.

Initially, the adopted treatment to eradicate *H. pylori*, the so-called ‘legacy’ triple therapy with a proton-pump inhibitor (PPI), Clarithromycin and Amoxicillin was quite effective. However, in the last 10 years, its’ efficacy has decreased to unacceptable levels in many countries, especially due to increasing levels of bacterial resistance [3]. Excessive and misjudged use of antibiotics contributed to this decreased susceptibility, leading to 50% resistance rates to Clarithromycin in the central region of Portugal (primary 21.4%; secondary 88.3%) [4]. Accordingly, current guidelines recommend bismuth-containing quadruple therapies or, alternatively, sequential or non-bismuth quadruple therapy, if Clarithromycin resistance is above the 15–20% threshold [5]. Unfortunately, *H. pylori* antibiotic resistance has reached alarming levels not only for macrolides but also to other drugs. Metronidazole resistance has been stable in Europe, reaching 34.9% of *H. pylori* isolates. Fortunately, its impact on the success of eradication regimens is limited, as it can be overcome by prolonging duration of treatment or increasing the dosage of Metronidazole [6]. Despite obtaining adequate eradication rates, standard sequential treatment should not be prescribed if the combined Clarithromycin and Metronidazole resistance is over 5% (which is the case in several European countries) [6]. To overcome these problems, some physicians prefer prescribing an adapted sequential treatment with Levofloxacin instead of Clarithromycin, as quinolones have been suggested as an alternative in both first- and second-line regimens [7]. Patients with reported history of penicillin allergy were also often prescribed with such regimens. Regrettably, resistance rates to quinolones also reached worrisome figures in Portugal and in Europe, reflecting its ubiquitous use for minor respiratory and urinary infections. According to Almeida *et al* [4], overall resistance to Levofloxacin in the center of Portugal is currently 33.9% (primary: 26.2%; secondary: 44.2%). Facing such adverse ecological scenario, our aim was to compare the efficacy of Clarithromycin and Levofloxacin-based sequential quadruple therapies as first-line treatment options and determine factors associated with treatment failure.

## Methods

### Study design

This retrospective study was conducted at the Coimbra University Hospital from January 2014 to December 2015. Adult patients with infection by *H. pylori* defined by histology or ^13^C-urea breath test (UBT) and treated with either standard sequential treatment (SST; group A) or Levofloxacin-based sequential treatment (LST; group B) were considered for inclusion. The treatment regimen was chosen according to their physicians’ preference. Other inclusion criteria were: (1) age over 18 years-old, (2) absence of known allergies for antibiotics used in either regimen, (3) signed informed consent. A total of 358 patients treated with one of these regimens were interviewed after treatment completion (group A – 250 patients; group B – 108 patients). Collected data included age, gender, smoking and alcohol consumption, indication for treatment, side effects and adherence. In order to obtain two groups with similar dimension, and therefore avoid statistical bias, we opted to include 100 patients in each group. The selection of patients was performed randomly, using SPSS software (as detailed below). Eradication therapy was prescribed by Gastroenterologists or other physicians working in our hospital.

The exclusion criteria were: (1) patients with previous *H. pylori* eradication attempts; (2) patients treated with proton-pump inhibitor (PPI), H_2_ receptor antagonist or antibiotics in the 4 to 6 weeks previous to UBT; (3) patients that interrupted the treatment before completion for any reason; (4) non-adherence to treatment. The stricter definition of non-adherence was used, and patients that failed at least one dose of medication were excluded.

All patients were submitted to UBT at least 2 months after completing treatment. The primary outcome of the study was the eradication rate of *H. pylori* infection with SST and LST. Finding factors associated with treatment failure was the secondary outcome.

### Therapeutic regimens

Patients in group A were treated with standard sequential therapy (SST) for 10 days: PPI twice daily + Amoxicillin 1 g twice daily for 5 days; followed by PPI twice daily + Clarithromycin 500 mg twice daily + Metronidazole 500 mg twice / thrice daily or Tinidazole 500 mg twice daily for five more days. Group B patients were treated with Levofloxacin-containing sequential therapy (LST) for 10 days: PPI twice daily + Amoxicillin 1 g twice daily for 5 days followed by PPI twice daily + Levofloxacin 250 mg twice daily + Metronidazole 500 mg twice / thrice or Tinidazole 500 mg twice daily for five more days. The choice between different PPI’s, Metronidazole or Tinidazole and its dosage was made by the prescribing physician.

### Statistical analysis

Statistical analysis of the results was performed using chi-square test, Student’s *t*-test and Fisher’s exact test as well as binomial logistic regression for multivariate analysis. For all analysis, P values <0.05 were considered significant. The 95% confidence intervals (CIs) were calculated by normal approximation. The analysis was performed using SPSS for Windows (version 21; SPSS Inc.).

## Results

### Study population

These groups were compared regarding demographic and clinical characteristics, as described in Table 1. There was no significant age or gender difference between both groups. The same can be said for smoking habits and alcohol consumption (defined as more than 10 cigars/day and >20 g alcohol each day). The main indication for prescribing eradication therapy was non-ulcer dyspepsia in both groups. First-generation and full-dose PPI were preferred in both the SST and the LST regimens. There were significant differences in the choice of the nitroimidazole antibiotics: Tinidazole was prescribed more often in both groups, but especially in group B (*p* = 0.001). The most used dosage for Metronidazole was 500 mg three times a day in both groups. The majority of the prescribing physicians were Gastroenterologists, especially in group B (*p* = 0.002).

**Table 1:** Patients’ demographic and clinical characteristics

	Group A (SST) *n* = 100	Group B (LST) *n* = 100	*p*
Mean age (years)	52.4 ± 16.2 (range 18–89)	54 ± 15.3 (range 18–81)	0.458
Gender			
- Female - Male	55% 45%	60% 40%	0.567
Smoking habits	8%	8%	1
Alcohol consumption	19%	10%	0.107
Indication(s) for *H. pylori* eradication			
- Non-ulcer dyspepsia - Peptic ulcer - GERD/chronic use of PPI - Before bariatric surgery - Anemia or thrombocytopenia - Familial history of gastric cancer	63% 14% 11% 9% 2% 1%	72% 10% 11% --- 7% ---	0.307 0.452 1 --- 0.229 ---
Nitroimidazole antibiotic			
- Tinidazole - Metronidazole	53% 47%	81% 19%	0.001
Metronidazole dosage			
- 500 mg 12–12 h (1000 mg) - 500 mg 8–8 h (1500 mg)	29,8% 70,2%	21,1% 79,9%	0.554
PPI 1^st^ or 2^nd^ generation			
- Omeprazole / Pantoprazole / Lansoprazole - Esomeprazole / Rabeprazole	81% 19%	89% 11%	0.165
PPI dosage			
- Full-dose - Half-dose	91% 9%	88% 12%	0.645
Prescribing Physician			
- Gastroenterologist - Non-Gastroenterologist	79% 21%	94% 6%	0.002

*SST* standard sequential treatment, *LST* levofloxacin-based sequential treatment, *GERD* gastroesophageal reflux disease, *PPI* proton-pump inhibitor

### Efficacy of eradication therapy

The overall eradication rate was 84.5% (169/200). The eradication rates were 90% (90/100) for group A and 79% (79/100) for group B. Standard sequential therapy was more effective than Levofloxacin-based modified sequential therapy (*p* = 0.03; OR = 2.39; IC95% = 1.06–5.38).

### Factors associated with eradication failure

In group A, the use of Metronidazole instead of Tinidazole (*p* = 0.006; OR = 10.15; IC95%:1.34–77.15), a lower dose of Metronidazole (*p* = 0.001; OR = 8.25; IC95% = 1.95–34.81) and not using an adequate dosage of PPI (*p* = 0.001; OR = 10.11; IC95% = 3.60–28.41) were risk factors for eradication failure – as described in Table 2. Multivariate analysis confirmed that half-dose PPI (*p* = 0.001; OR = 16.25; IC95% = 2.91–89.21) and a lower dose of Metronidazole (*p* = 0.007; OR = 13.58; IC95% = 2.07–89.04) were associated with unsuccessful eradication (Table 2). In group B, half-dose PPI and prescription from non-Gastroenterologists were the two factors associated with unsuccessful eradication (*p* = 0.001; OR = 3.67; IC95% = 1.86–7.22 and *p* = 0.017; OR = 3.68; IC95% = 1.81–7.50, respectively). Only the use of half-dose PPI was associated with unsuccessful eradication in multivariate analysis (*p* = 0.011; OR = 5.69; IC95% = 1.48–21.90).

**Table 2:** Potential risk factors associated with eradication failure

	Group A (*n* = 100) Standard Sequential Therapy			Group B (*n* = 100) Levofloxacin-based Sequential		
	Success (*n* = 90)	Failure (*n* = 10)	p/OR (95%CI)	Success (*n* = 79)	Failure (*n* = 21)	p/OR (95%CI)
Age	52.1 ± 1.8	55,5 ± 3,8	0.528	55.7 ± 1.6	47.7 ± 3.9	0.068
>65 years	23 (25.6%)	2 (20%)	0.720	23 (29.1%)	7 (33.3%)	0.708
Female	50 (55.6%)	5 (50%)	0.738	49 (62%)	11 (52.4%)	0.423
Smoking habits	6 (6.7%)	2 (20%)	0.182	5 (6.3%)	3 (14.3%)	0.359
Alcohol consumption	16 (17.8%)	3 (30%)	0.350	8 (10.1%)	2 (9.5%)	0.952
Non-ulcer dyspepsia	14 (17.9%)	0	0.353	63 (83.3%)	20 (95.2%)	0.112
Metronidazole (vs. Tinidazole)	38 (42.2%)	9 (90%)	0.006/10.15 (1.34–77.15)	16 (20.3%)	3 (14.3%)	0.756
Metronidazole 1000 mg (vs. 1500 mg)	7 (7.8%)	7 (70%)	0.001/ 8.25 (1.95–34.89)	4 (25%)	0	0.964
1^st^ Generation PPI (vs. 2^nd^ Generation)	74 (82.2%)	7 (70%)	0.396	69 (87.3%)	20 (95.2%)	0.450
Half-dose PPI (vs. full-dose)	4 (4.4%)	5 (50%)	0.001/ 10.11 (3.60–28.41)	5 (6.3%)	7 (33.3%)	0.001/3.67 (1.86–7.22)
Prescription by non-Gastroenterologist	20 (22.2%)	1 (10%)	0.684	2 (2.5%)	4 (19%)	0.017/3.68 (1.81–7.50)

*CI95%* 95% confidence interval, *OR* odds ratio, *PPI* proton-pump inhibitor

## Discussion

In southern European countries, such as Portugal, with alarming resistance rates to most commonly used antibiotics, recommending an effective first-line treatment for *H. pylori* eradication is becoming increasingly difficult. Taking into account resistance rates to Clarithromycin of over 30%, an alternative quadruple regimen with Levofloxacin could be an effective option. However, with a resistance rate to Levofloxacin over 25%, its utility may be compromised. But if the resistance rates to Clarithromycin and Levofloxacin are similar, why did the two therapies showed such different results? Probably this is due to the interaction between Amoxicillin and Clarithromycin. Amoxicillin acts on the bacterial wall, destroying a transmembrane efflux system that allows elimination of Clarithromycin in resistant bacterium. Therefore there is a higher intracellular concentration of Clarithromycin which can overcome other resistance mechanisms [8]. The first 5 days of Amoxicillin act as an inductor for the following 5 days of Clarithromycin. This is probably the reason behind the ability of the sequential regimen to eradicate a significant percentage of Clarithromycin-resistant strains (as high as 82% according to Zullo *et al* [9]). Interestingly, our results are quite different from those obtained in a randomized trial conducted in Italy, where Levofloxacin-based sequential regimens obtained >95% eradication rates, while a Clarithromycin-based sequential regimen obtained only an 80.8% success rate [10]. Other studies showed the same superiority for Levofloxacin-based triple and sequential regimens [11–14]. A recently published meta-analysis compared Levofloxacin-based sequential therapy and Clarithromycin-based triple and sequential treatment, and revealed superiority for the regimens that included fluoroquinolones (87.8% vs. 71.1%, respectively) [15]. This is probably due to the very low resistance to Levofloxacin reported in such studies (as low as 3.7% in the Italian study by Romano *et al* [10]).

The patients included in our study were treated with Metronidazole 500 mg in an 8–8 h or 12–12 h or with Tinidazole 500 mg 12–12 h. Tinidazole was chosen more often in both groups, but this preference was only significant in the LST group. When comparing both antibiotics, Metronidazole was inferior to Tinidazole in the SST group. This difference is probably due to the use of lower doses of Metronidazole (1000 mg) in almost a quarter of the patients. It is known that resistance to Metronidazole in southern European countries is over 30% [4, 16], but can be overcome by using longer treatments or higher doses of the drug. This hypothesis is supported by the fact that high-dose Metronidazole (1500 mg) obtained similar results to Tinidazole. This difference in efficacy when using different Metronidazole dosages was not observed in the LST group, probably due to the low number of patients treated with Metronidazole (19%), not allowing for statistically significant differences to be noted.

When facing high resistance levels to Metronidazole, it has been suggested that the optimum duration of quadruple therapies should be 14 days, combined with high doses of the drug (up to 1500–1600 mg in divided dosages) [17]. Despite being recommended to avoid 10-day sequential treatment if Metronidazole resistance is over 20%, in our population a 90% eradication rate was obtained nevertheless.

Besides antibiotic resistance, there are other factors that may contribute to treatment failure. Smoking is often associated with unsuccessful eradication [18, 19], but this was not the case in our study, probably due to the low number of smokers included in our sample population. Non-ulcer dyspepsia patients also tend to respond worse to eradication treatment, when compared to those with peptic ulcers [20], but this tendency was not observed in our study groups. There was also no difference in eradication rates for patients submitted to treatment for hematologic conditions such as anemia or thrombocytopenia. In fact, the most consistent factor that influenced eradication success for both groups was the proton-pump inhibitor (PPI) dose. The crucial role of acid inhibition in optimizing the action of antibiotics such as Amoxicillin led to the recommendation of twice-daily full-dose PPI regimen [5, 21]. While the use of full-dose PPI is widely recommended, the need to use more recent PPI’s instead of first-generation drugs is more controversial. A meta-analysis by McNicholl *et al* [22], revealed superiority of esomeprazole and rabeprazole, but other studies failed to show the same benefit [23]. Using new-generation PPI’s may be more beneficial in populations where CYP2C19 extensive metabolizer phenotype is prevalent [24]. In our population the use of new-generation PPI’s was not associated with a higher eradication rate. Another interesting finding was the difference in the eradication rates obtained by Gastroenterologists and non-Gastroenterologists in the LST group. While there is no obvious explanation for this difference, it has been suggested that providing the patient with a written plan with prescription details and warning about expected adverse effects may improve adherence and therefore may contribute to eradication success [25, 26].

Our study has some limitations. The strict non-adherence definition used in our study led to the exclusion of a significant number of patients. Furthermore, to obtain two groups with similar dimension, a randomized sampling of patients was performed. Despite these constraints, our study groups’ size is within range when compared to other published studies [4, 12, 16, 25]. Its’ retrospective nature does not allow comparison in intention-to-treat and per-protocol analysis and *H. pylori* antibiotic resistance was not determined for each included patient.

## Conclusions

We can conclude that standard sequential treatment was quite effective and achieved an eradication rate that reached the threshold of 90%, despite Metronidazole resistance over 20% and simultaneous resistance to Clarithromycin and Metronidazole over 5% in our country. Full-dose PPI and a higher dose of Metronidazole should be adopted to insure positive outcomes. Levofloxacin-based sequential regimen achieved an eradication rate under 80% and should not be considered as first-line therapy.

To achieve such results with standard sequential therapy, a full-dose of PPI and high doses of Metronidazole or Tinidazole should be used. Despite adequate success rate demonstrated by Clarithromycin-based sequential therapy, a prospective study comparing different non-bismuth quadruple therapies (sequential, hybrid and concomitant) should be conducted in the near future to find out which is the most effective in a country with high Clarithromycin and Levofloxacin resistance such as Portugal.

## Additional file

Additional file 1: Supplementary material. Set of data from all patients included in the study (SPSS file). (XLS 105 kb)

## Abbreviations

BID/TID: Two/three times a day
CI95%: 95% confidence interval
GERD: Gastroesophageal reflux disease
LST: Levofloxacin-based sequential treatment
OR: Odds ratio
PPI: Proton-pump inhibitor
SST: Standard sequential treatment
UBT: Urea breath test

## References

[1] Eusebi LH, Zagari RM, Bazzoli F (2014). Epidemiology of Helicobacter pylori infection. Helicobacter.

[2] Roubaud Baudron C, Franceschi F, Salles N, Gasbarrini A (2013). Extragastric diseases and Helicobacter pylori. Helicobacter.

[3] Graham DY, Fischbach L (2010). Helicobacter pylori treatment in the era of increasing antibiotic resistance. Gut.

[4] Almeida N, Romãozinho JM, Donato MM, Luxo C, Cardoso O, Cipriano MA (2014). Helicobacter pylori antimicrobial resistance rates in the central region of Portugal. Clin Microbiol Infect.

[5] Malfertheiner P, Megraud F, O'Morain CA, Atherton J, Axon AT, Bazzoli F (2012). Management of Helicobacter pylori infection — the Maastricht IV / Florence consensus report. Gut.

[6] Megraud F, Coenen S, Versporten A, Kist M, Lopez-Brea M, Hirschl AM (2013). Helicobacter pylori resistance to antibiotics in Europe and its relationship to antibiotic consumption. Gut.

[7] Gisbert JP, Morena F (2006). Systematic review and meta-analysis: levofloxacin-based rescue regimens after Helicobacter pylori treatment failure. Aliment Pharmacol Ther.

[8] De Francesco V, Margiotta M, Zullo A, Hassan C, Troiani L, Burattini O (2006). Clarithromycin-resistant genotypes and eradication of Helicobacter pylori. Ann Intern Med.

[9] Zullo A, De Francesco V, Hassan C, Morini S, Vaira D (2007). The sequential therapy regimen for Helicobacter pylori eradication: a pooled-data analysis. Gut.

[10] Romano M, Cuomo A, Gravina AG, Miranda A, Lovene MR, Tiso A (2010). Empirical Levofloxacin-containing versus Clarithromycin-containing sequential therapy for Helicobacter pylori eradication: a randomised trial. Gut.

[11] Molina‐Infante J, Perez‐Gallardo B, Fernandez‐Bermejo M, Hernandez‐Alonso M, Vinagre G, Duenas C (2010). Clinical trial: clarithromycin vs. levofloxacin in first-line triple and sequential regimens for Helicobacter pylori eradication. Aliment Pharmacol Ther.

[12] Polat Z, Kadayifci A, Kantarcioglu M, Ozcan A, Emer O, Uygun A (2012). Comparison of Levofloxacin-containing sequential and standard triple therapies for the eradication of Helicobacter pylori. Eur J Intern Med.

[13] Qian J, Ye F, Zhang J, Yang YM, Tu HM, Jiang Q (2012). Levofloxacin-containing Triple and Sequential Therapy or Standard Sequential Therapy as the First Line Treatment for Helicobacter pylori Eradication in China. Helicobacter.

[14] Lee H, Hong SN, Min BH, Lee JH, Rhee PL, Lee YC (2015). Comparison of efficacy and safety of Levofloxacin-containing versus standard sequential therapy in eradication of Helicobacter pylori infection in Korea. Dig Liver Dis.

[15] Kale‐Pradhan PB, Mihaescu A, Wilhelm SM (2015). Fluoroquinolone sequential therapy for helicobacter pylori: a meta-analysis. Pharmacotherapy.

[16] Georgopoulos SD, Xirouchakis E, Martinez‐Gonzalez B, Sgouras DN, Spiliadi C, Mentis AF (2013). Clinical evaluation of a ten-day regimen with esomeprazole, metronidazole, amoxicillin, and clarithromycin for the eradication of helicobacter pylori in a high clarithromycin resistance area. Helicobacter.

[17] Graham DY, Lee SY (2015). How to effectively use bismuth quadruple therapy: the good, the bad, and the ugly.

[18] Kim SE, Park MI, Park SJ, Moon W, Choi YJ, Cheon JH (2015). Trends in Helicobacter pylori eradication rates by first-line triple therapy and related factors in eradication therapy. Korean J Intern Med.

[19] Pan KF, Zhang L, Gerhard M, Ma JL, Liu WD, Ulm K, et al. A large randomised controlled intervention trial to prevent gastric cancer by eradication of Helicobacter pylori in Linqu County, China: baseline results and factors affecting the eradication. Gut. 2015; Gutjnl. 10.1136/gutjnl-2015-309197 25986943

[20] Wolle K, Malfertheiner P (2007). Treatment of Helicobacter pylori. Best Pract Res Clin Gastroenterol.

[21] Villoria A, Garcia P, Calvet X, Gisbert JP, Vergara M (2008). Meta-analysis: high-dose proton pump inhibitors vs. standard dose in triple therapy for Helicobacter pylori eradication. Aliment Pharmacol Ther.

[22] McNicholl AG, Linares PM, Nyssen OP, Calvet X, Gisbert JP (2012). Meta-analysis: esomeprazole or rabeprazole vs. first-generation pump inhibitors in the treatment of Helicobacter pylori infection. Aliment Pharmacol Ther.

[23] Choi HS, Park DI, Hwang SJ, Park JS, Kim HJ, Cho YK (2007). Double-dose, new-generation proton pump inhibitors do not improve Helicobacter pylori eradication rate. Helicobacter.

[24] Georgopoulos SD, Papastergiou V, Karatapanis S (2015). Treatment of Helicobacter Pylori infection: optimization strategies in a high resistance era. Expert Opin Pharmacother.

[25] Lee M, Kemp JA, Canning A, Egan C, Tataronis G, Farraye FA (1999). A randomized controlled trial of an enhanced patient compliance program for helicobacter pylori therapy. Arch Intern Med.

[26] Al‐Eidan FA, McElnay JC, Scott MG, McConnell JB (2002). Management of Helicobacter pylori eradication--the influence of structured counselling and follow-up. Br J Clin Pharmacol.

